# Electrostatic Dust Cloth: A Useful Passive Sampling Method When Assessing Exposure to Fungi Demonstrated in Studies Developed in Portugal (2018–2021)

**DOI:** 10.3390/pathogens11030345

**Published:** 2022-03-12

**Authors:** Carla Viegas, Marta Dias, Susana Viegas

**Affiliations:** 1 H&TRC—Health & Technology Research Center, ESTeSL—Escola Superior de Tecnologia da Saúde, Instituto Politécnico de Lisboa, 1990-096 Lisbon, Portugal; marta.dias@estesl.ipl.pt (M.D.); susana.viegas@ensp.unl.pt (S.V.); 2 Public Health Research Centre, NOVA National School of Public Health, Universidade NOVA de Lisboa, 1099-085 Lisbon, Portugal; 3 Comprehensive Health Research Center (CHRC), NOVA Medical School, Universidade NOVA de Lisboa, 1169-056 Lisbon, Portugal

**Keywords:** EDC, microbiological contamination, passive sampling method, indoor environments, occupational exposure assessments

## Abstract

Electrostatic dust cloths (EDC) have been widely used for microbiologic contamination assessment in different indoor and occupational environments. This paper reviews sixteen studies performed in Portugal between 2018 and 2021 for evaluating the exposure to microbiological agents and focusing on fungi using EDC as a passive sampling method. The findings suggest that EDC can be applied as a screening method for particulate matter-exposure assessment and as a complementary method to characterize microbial exposures in occupational environments. Overall, EDC should be included, side by side with other sampling methods, in sampling campaigns focused on exposure assessments due to the advantages such as the straightforward extraction protocol favoring the employment of different assays, which allows us to assess exposure to a wide range of microbial agents, and presents higher accuracy regarding the fungal diversity.

## 1. Introduction

### 1.1. Exposure Assessment and the Use of Electrostatic Dust Cloths

Current sampling strategies used for microbial exposure assessment may ineffectively describe significant exposures. Even if we apply the state-of-the-art regarding analyses, the information can be biased if our sampling techniques are not properly selected [1]. Thus, it is critical to select the best sampling approach to allow the accurate measurement and identification of the microbiological agents present in the indoor environments to be assessed.

In a recent study performed by Adams et al. (2021) [2] in a school’s environment and using electrostatic dust collectors (EDC) it was possible to identify the microorganisms related to inspection-based building moisture damage and then examine the links between those microbial exposures and health effects [2]. Indeed, this sampling method has been widely used for microbiologic contamination assessment in different indoor and occupational environments (Table 1). If the intention is to perform viability studies, the electrostatic cloth used should not be impregnated with any kind of biocide to avoid impairing the viability of the microorganisms viability.

This sampling method is appropriate for large-scale epidemiological studies intending to measure microbial exposure, and to complement exposure information collected by building inspections dedicated to spotting dampness and mold [3,4]. EDC is also being used to assess microbial contamination using molecular tools as stand-alone analyses [4,5,6,7,8], even the most refined ones such as sequencing [2], or using side by side, culture-dependent, and independent methods [3,9,10,11,12,13,14,15,16,17,18,19,20,21,22,23]. In fact, the use of qPCR analyses from EDC is a promising tool to accurately measure microbial contamination in dwellings [4,8,24]. Additionally, it has also been used to perform the fungal azole resistance screening in different indoor environments [12,13,14,15,16,17,18,19,20,21,22,23,24,25,26] and to identify MRSA presence and level of contamination in specific occupational environments [7]. Further, aside from microbial contamination, this sampling method has been applied to assess microbial metabolites, such as endotoxins [27,28,29] and mycotoxins [18,22]. Furthermore, the EDC was also used as a sampling method to measure antigen concentrations with enzyme immunoassays specific for storage mites [30].

### 1.2. Electrostatic Dust Cloths’ Features

As all passive sampling methods, it allows a more integrated time exposure assessment (workshift, days, weeks, or months), since it can collect during different periods of time depending on the activities, work shifts, and expected contamination [2,15,17,18]. In fact, this sampling method can be applied for prolonged periods of time, and, because of that, they allow us to overcome the major drawback of short-term active air sampling, which is highly sensitive to large temporal fluctuations in the airborne microbial load that might be associated to specific events that occur only sporadic in a specific workplace or indoor environment [4]. They have a very low cost (petri dish and an electrostatic cloth) and do not require microbiological training to set up and can be applied by the study subjects themselves in their dwellings [4]; however, in the workplaces, the EDC should be placed by a trained technician to select the proper sampling sites considering the study aim and the most suitable surfaces (preferably elevated surfaces at the height of 1.5–2.5 m) avoiding sampling sites with major airflow disturbances [2,15,17,18]. EDC placed on an elevated surface, besides collecting particles over a known time period, allows capturing airborne dust instead of floor-based particles that may never become sufficiently airborne to contribute to human exposure by inhalation [6]. Previously, a study performed by Madsen and colleagues (2012) [3] reported the need to place the EDC on open surfaces during sampling and that obtained can be frozen at −80 °C with glycerol without disturbing the microorganisms’ number considerably [3].

## 2. Studies Performed in Portugal

This paper reviews several studies performed in Portugal between 2018 and 2021 for assessing the exposure to microbiological agents using EDC as a passive sampling method (Table 1). The conducted studies were exploratory, since they were the first efforts to assess exposure to microorganisms in different Portuguese indoor environments. In fact, different indoor environments were assessed: veterinary clinic, bakeries, dwellings, health care facilities (including ambulances for patients ‘transportation), and firefighters’ headquarters (FFH).

Fifteen studies were included in the table, two of them being dedicated specifically to the use of EDC, aiming to investigate the adequacy of this device for characterizing the distribution patterns and exposure concentrations of particulate matter and microbial contaminants [10,15]. In both of them, the suggested procedures reported previously [3] were followed.

EDC was applied only with one other sampling method in the studies performed in dwellings to avoid disturbance in the occupant’s routine. In fact, from the four studies performed in dwellings, two filters of 47 mm diameter quartz fiber were also used to collect particulate matter [17,23]. In the remaining two, in one, PM2.5 and PM2.5–10 were measured with a medium volume sampler [16], and in the other, active sampling by impaction method was also performed [20].

EDC was one among the several sampling methods applied in the other studies reported in Table 1, presenting, in most of the studies, a higher number of passive methods than active sampling methods employed. In fact, besides being used in parallel with devices that allow air sampling, other different passive sampling methods were employed, such as surface swabs and settled dust and different environmental matrices (e.g., filters from HVAC, mops, cleaning cloths, uniform ranks, and identification badges) were collected depending on the indoor environment/setting being assessed [11,12,13,14].

The sampling period used for the EDC varied between studies. Fifteen days of sampling was followed in the two studies dedicated to bakeries, while in the other occupational environments, 30 days were applied. This difference was due to the expected microbial contamination in the assessed indoor environments. In the assessed dwellings, some constraints were faced due to the occupant’s availability when the sampling period ended, and although 30 days of sampling was to be followed, an extended period of sampling was performed in some cases. This difficulty was overcome by applying a different formula,

(CFU.m-2.day =(1 x/(3.14*EDC area))/days of sampling)(1)

where the sampling days were considered, to obtain fungal densities [21]. This different approach for the fungal density’s quantification was the one followed after the first study performed in dwellings [16].

The protocol used was common to all studies where the EDC was employed in the dedicated sampling campaigns and following the procedures previously published [3] and as follows: Each EDC cloth was washed with 20 mL 0.9% NaCl with 0.05% Tween80™ (Merck S.A, Lisbon, Portugal) by orbital shaking (250 rpm, 60 min, at room temperature), and 150 µL of the wash suspension was inoculated on to two different culture media: 2% malt extract agar (MEA) with 0.05 g/L chloramphenicol media and dichloran glycerol (DG18) agar-based media. After incubation of the plates with the selected media, bacteria and/or fungal densities were determined.

In all studies, the fungal contamination was characterized focusing on *Aspergillus* genera due to clinical and toxicological relevance from the *Aspergillus* sections [12,21], EDC provided information concerning fungal azole resistance in all the performed studies presented in Table 1, unveiling more data regarding this public and occupational health threat. Additionally, it was also possible to focus on *Aspergillus* sections indoors [12,21], as well as other fungal species, providing a more complete fungal characterization with a wider number of different fungal species being identified than the other active and passive sampling methods [13,14,17,18,20,31]. Although the bakeries setting presented the highest fungal contamination due to the role as contamination sources of the raw materials [10,14], the setting presenting the highest *Aspergillus* sp. contamination was the FFH due to the observed buildings damage and leakages [21]. Mycotoxin’s detection [18,22] and cytotoxicity assessment using different cell lines [13,18,23] were also assays employed in enlarged studies dedicated to microbiologic agents.

Almost all the studies used culture-based methods and qPCR for the detection of *Aspergillus* sections. The exception, where only culture-based methods were applied, was in the study performed in 12 bakeries [10], the study performed in different settings where molecular identification was achieved [31], and a study concerning *Aspergillus* section *Fumigati* where the isolates were recovered by culture-based methods for further analyses [23]. In the studies where culture-based methods and qPCR detection were applied side by side, complementary results were obtained with higher detection of *Aspergillus* sections, mainly section *Fumigati*, by molecular tools.

**Table 1:** Studies performed in Portugal to assess indoor exposure to microbial contamination by using EDC.

Indoor Environment	Study Goal	Sampling Methods	Analyses Performed to EDC	Most Relevant EDC Fungal Results	Main Conclusions	Reference
One veterinary clinic	Assessment of organic dust and microbial contamination in a typical Portuguese veterinary clinic, including azole-resistant fungi.	Active (air impaction N = 8) and passive (surfaces N = 8 and **EDC N = 3**)	Culture-based methods (fungi and bacteria and azole resistance screening) and qPCR (*Aspergillus* sections detection)	EDC results evidenced the presence of *Fusarium equisetii* and *Cladosporium* sp. on MEA and DG18, respectively.	The sampling protocol in veterinary clinics should comprise active and passive sampling methods. Culture-dependent and independent methods should be used to achieve a more complete characterization of the microbial contamination.	[11]
Twelve bakeries	To analyze the adequacy of EDC for identifying the distribution patterns and exposure concentrations of particulate matter and microbial contaminants in bakeries.	Passive sampling method (**EDC N = 33**) and Particle counts and size distribution (0.3 µm, 0.5 µm, 1 µm, 2.5 µm, 5 µm, and 10 µm) measurement	Culture-based methods (bacteria and fungi)	Higher EDC mass was significantly correlated with higher fungal load on DG18 and with particle size distribution in different dimensions *Penicillium* sp. (42.56%) was the most frequent fungi.	EDC was useful for identifying critical workplaces regarding exposure to particulate matter and microbial contamination. Results obtained suggest that EDC can be applied as a screening method in exploratory studies concerning particulate matter-exposure assessment and to quantify exposures in specific occupational environments.	[10]
Thirteen bakeries	To assess workers´ exposure to fungi and mycotoxins in Portuguese bakeries.	Active methods (Air impaction and impingement each N = 53) and passive (surface swabs N = 58, **EDC N = 36** and settled dust N = 11) methods	Culture-based methods (fungi) and qPCR (*Aspergillus* sections)	*A.* section *Fumigati* presented 50% of prevalence on DG18. it was possible to detect section *Fumigati* in 7.4% on EDC samples	A wide number of sampling methods (active and passive) and different assays (culture-based and molecular methods) should be employed to obtain a refined risk characterization regarding fungi and mycotoxins.	[14]
Ten Primary Health Care Centres (PHCC)	*Aspergillus* distribution assessment in 10 PHCC	Active (impaction N = 81 and impingement N = 41) and passive (surface swabs N = 81, **EDC N = 81**, settled dust N = 10, and filters from HVAC system N = 12) methods	Culture-based methods (*Aspergillus* prevalence) and qPCR (*Aspergillus* sections)	*Fumigati* section presented 1.3% of prevalence on EDC	One PHCC was not in compliance with IAQ Portuguese law since *Aspergillus* section *Fumigati* counts surpassed the quantitative guideline. The results of this study show that *Aspergillus* is widely spread in PHCC. The use of a multi-approach sampling protocol with selective media should be the trend in regular assessments performed in clinical environments.	[12]
	To analyze the adequacy of EDC for identifying critical workstations of occupational exposure to particulate matter and for characterizing the microbial contamination present in 10 PHCC.	Particle counts and size distribution were measured with direct-reading equipment. Passive sampling method (**EDC N = 81**)	Culture-based methods (bacteria and fungi) and qPCR (*Aspergillus* sections)	In MEA *A.* section *Fumigati* was observed in smaller counts (0.01%) and it was identified in the cleaning supply room.	The EDC was useful for unveiling the microbial contamination on the assessed PHCC.	[15]
One Central Hospital from Oporto	To assess the exposure to bioburden in one central hospital with a multi-approach protocol using active and passive sampling methods.	Active methods (impaction N = 120, filtration N = 2, and impingement N = 15) and passive (surface swabs N = 45, **EDC N = 15** and settled dust N = 5; HVAC filter samples N = 2)	Culture-based methods (fungi and bacteria and azole resistance screening) and qPCR (*Aspergillus* sections). Mycotoxins and endotoxins profile were also assessed. Two cytotoxicity assays were conducted with two cell lines and in vitro pro-inflammatory potential was assessed	*Fumigati* section was observed in all the samples where culture-independent tools were applied including EDC (100%, 15 samples out of 15).	A multi-approach concerning sampling and analysis methods should be applied in the hospital environment	[13]
Different occupational and nonoccupational indoor settings	Molecular identification of *Aspergillus* species collected	Several environmental matrices depend on the indoor environment. **EDC was used in 4 out of the 7 environments** assessed	Culture-based methods *(Aspergillus* and azole resistance screening from the isolates) and molecular identification	Five *Aspergillus* isolates obtained from EDC were selected for further analyses (*A. welwitschiae* (n = 2), *A. tubingensis* (n = 1), *A. fumigatus* sensu stricto (n = 1) and *A. clavatus* sensu stricto (n = 1))	Resistance profile of *Aspergillus* in specific indoor environments was characterized. *Aspergillus* epidemiology characterization allows a complete risk characterization concerning *Aspergillus* burden.	[31]
One Central Hospital from Lisbon	Bioburden assessment with two passive sampling methods (ventilations grids swabs and electrostatic dust collectors (EDC) at Clinical Pathology Services.	Surface swabs (N = 30) from ventilation grids and **EDC (N = 16)**	Culture-based methods (fungi and bacteria and azole resistance screening) and qPCR (*Aspergillus* sections). Mycotoxins assessment and cytotoxicity profile was also performed	*Aspergillus* section *Fumigati* was detected in 7 EDC samples (7 out of 12; 58.33%).	The use of the two sampling methods—swabs and EDC—allowed us to obtain a more complete characterization of the microbial contamination. Culture-dependent and independent methods used side by side allow to perform an accurate characterization of the *A.* section *Fumigati* contamination.	[18]
Thirty-three dwellings and four schools	To assess microbial contamination in the indoor microenvironments more frequented by children	PM2.5 and PM2.5–10 was sampled with a medium volume sampler. EDC was placed in the living room (**N = 33**) and in the children’s bedroom (N = 31), and in schools (**N = 4**)	Culture-based methods (fungi and bacteria and azole resistance screening) and qPCR (*Aspergillus* sections)	The fungal species most frequently found in bedrooms was *Penicillium* sp. (91.79%), while, in living rooms, it was found *Rhizopus* sp. (37.95%) was the most prevalent. *Aspergillus* sections with toxigenic potential were observed in bedrooms and living rooms and were able to grow on VOR.	Future studies, applying EDC sampling method coupled with PM assessment, should be performed to allow for a long-term integrated sample of organic dust.	[16]
Twenty-three dwellings	To assess settleable dust loading rates and microbial contamination in Portuguese dwellings by passive sampling (quartz fiber filters and EDC, respectively.)	Quartz fiber filters were placed side by side With EDC in summer (**N = 79**) and winter (**N = 78**)	Culture-based methods (fungi and bacteria and azole resistance screening) and qPCR (*Aspergillus* sections)	Dust and microbial contamination showed higher variability in the summer season. In both seasons, *Penicillium* sp. was the most prevalent (59.1% winter; 58.1% summer), followed by *Aspergillus* sp. in winter (13.0%). Fungal contamination increased in the winter period, whereas bacterial counts decreased. In the winter season, *Aspergillus* sections *Circumdati* and *Nidulantes* were detected in VOR supplemented media; *Aspergillus* sections *Fumigati* and *Nidulantes* were detected by molecular tools.	Passive sampling methods should be applied in sampling campaigns on dwellings. Azole resistance screening should be performed in dwellings, and culture-dependent and independent methods should be employed when assessing indoor microbial contamination.	[17]
Ten dwellings	Assessment of the bioburden during sleeping periods in Portuguese dwellings through active (air sampling) and passive (EDC) methods	Active sampling using a MAS-100™ air sampler equipment and EDC (from 7 bedrooms, 4 living rooms, and 1 kitchen) (**N = 12**)	Culture-based methods (fungi and bacteria and azole resistance screening) and qPCR (*Aspergillus* sections)	In bedrooms, the most found was *Penicillium* sp. (59.01% MEA; 96.54% DG18), in living room was *Aspergillus* section *Circumdati* on MEA (63.63%) and *Cladosporium* spp. on DG18 (100%), and in the kitchen was *Penicillium* sp. (100% MEA; 100% DG18). *Aspergillus* sp. in EDC ranged from 0 to 405.3 CFU/m^2^/day on MEA, while in DG18 *Aspergillus* species were not observed.	Bacterial increased during the sleeping period. Toxigenic fungal species and indicators of harmful fungal contamination, belonging to *Aspergillus* genera were identified indoors, as well as reduced susceptibility to antifungal drugs of some fungal species.	[20]
Thirty dwellings	To assess the deposition rates of total settleable dust and microbial contamination in the indoor air of dwellings onto quartz fiber filters andEDC, respectively	47 mm diameter quartz fiber filters were exposed to collect particulate matter and EDC (**N = 30**) were used for microbial contamination characterization	Culture-based methods (fungi and bacteria and azole resistance screening) and qPCR (*Aspergillus* sections)	Fungal contamination ranged from 1.97 to 35.4 CFU m^−2^ day^−1^ in MEA, and from undetectable to 48.8 CFU m^−2^ day^−1^ in DG18. *Penicillium* sp. was the most found in MEA (36.2%) and *Cladosporium* spp. in DG18 (39.2%).	Settleable dust and fungal contamination were increased in dwellings with pets; Indicators of harmful fungal contamination were present indoors; *Aspergillus* section *Candidi* was identified in azole supplemented media (VOR and POS); Specific housing typologies and characteristics influenced the microbial contamination.	[19]
Twelve ambulances vehicles	Assessment of the bioburden in Portuguese ambulances using active and passive sampling methods.	336 air samples through impaction method, 132 surface swabs, 7 mops, and cleaning cloths, 3 uniform ranks, 13 settled dust samples, and **14 EDC**	Culture-based methods (fungi and bacteria and azole resistance screening) and qPCR (*Aspergillus* sections) and mycotoxins detection	Fungal values ranged from 0 to 23 CFU m^−2^ day^−1^ in MEA, and 0 (to 28.3 CFU m^−2^ day^−1^ in DG18. *C. sitophila* was the most prevalent fungal species in EDC. Mycotoxins were detected in EDC.	EDC was useful for the fungal contamination characterization and also for mycotoxins detection on ambulances. Further studies are needed to determine the potential risk of infection transmission between different vehicles and under different conditions of use.	[22]
Eleven Firefighters headquarters (FFH)	Characterization of *Aspergillus* section *Fumigati* distribution in 11 firefighter headquarters (FFHs) to obtain an accurate occupational exposure assessment.	Active (air impaction method) (N = 760) and passive sampling methods (floor surfaces swabs (N = 90), electrostatic dust collectors (EDC) (**N = 82**), settled dust (N = 11), filters used for sampling the settled dust (N = 90), firefighter uniform badges (N = 67), cleaning cloths (N = 25) and mops N = 14).	Culture-based methods (fungi and azole resistance screening) and qPCR (*Aspergillus* sections)	*Aspergillus* genera was predominant in EDC from FFH4 (0.55%), whereas in FFH2, the section *Fumigati* was the most frequent in EDC (100%). The *Fumigati* section was predominant among *Aspergillus* genus in EDC samples in MEA (28.57%). Concerning azole resistance screening, *Aspergillus* genera were identified only in mops, EDC, settled dust filters, and settled dust. The *Fumigati* section was prevalent in the SDA of 4.4% in EDC and was the only section found in ITR (EDC: 100%) and the most observed in VOR (EDC: 97.1%). The same sectionwas detected by qPCR in almost all passive samples, with EDC being the sampling method with the highest prevalence (n = 61; 67.8%).	This study confirms the widespread of *Aspergillus* sp. in all FFHs. *Fumigati* section was identified in all FFHs as well as fungi potentially resistant to azoles.	[21]
Health Care Environments (10 Primary Health Care Environments (PHCC) and 1 Central Hospital (CH))	Cytotoxicity evaluation of *Aspergillus* section *Fumigati*	Active sampling (air sampling by impaction N = 201 andimpigment N = 56 for molecular detection purposes). Passive sampling (surface swabs–N = 126; **EDC, N = 96**; settled dust N = 15; and filters from HVAC system N = 12). Nasal swabs were collected from volunteer health care workers in the 10 PHCC (N = 25) and in the CH (N = 22).	*Aspergillus* Section *Fumigati* isolation by culture-based methods (including azole resistance screening) and cytotoxicity assessment in lung epithelial cells and kidney cells using the MTT assay.	1 isolate was recovered from EDC with a high level of cytotoxicity in both cell lines (A549 and SK cell lines)	Further studies should address the epidemiology and clinical relevance of *Aspergillus* section *Fumigati*, as well as the mechanisms underlying *Aspergillus*-mediated cytotoxicity.	[23]

## 3. Exposure Assessment: The Role and Advantages of the EDC

This study only focuses on studies performed in Portuguese indoor environments; however, several others using the sampling approach were mentioned, reporting also the features of the EDC as a sampling method. In addition, a previous review on the use of EDC was performed before this sampling method started to be used as a sampling resource in sampling campaigns [32]. EDC allows a wide array of assays to be employed afterward, featuring an enriched exposure assessment. This sampling method allows overcoming some of the limitations when facing the need to perform an accurate exposure assessment dedicated to fungi in the scope of indoor air quality or occupational health. Indeed, besides the features already mentioned, this sampling method has little impact on occupants/workers daily routine, being also suitable for enlarged studies dealing with several EDC and respective extracts. 

Since during EDC extraction, the recovery of microbial contamination can be partially lost (as in all sampling methods) [3], the recommendation is to use more than one sampling method in exposure assessments [33], being the EDC a suitable sampling method to complement active sampling methods, as well as to be used in parallel with more specific passive methods, that can be adjusted to the indoor environment under study (for instance filters from forklifters´ HVAC, identification badges from ambulances crew, …).

Although the trend is to apply more refined molecular tools to obtain the fungal diversity from the indoor environment to be assessed [2], since the viable part constitutes only a reduced part of the total composition [34] in exposure assessments culture-based methods are still needed to be applied to allow: (a) guidelines and legal framework comparison [12,21]; (b) to draw conclusions regarding the inflammatory potential variation, since the inflammatory and/or cytotoxic potential can affect the fungal viability [35,36] and; (c) to recover isolates for azole resistance screening, sequencing and mutations detection [31,37].

The findings also suggest that EDC can be applied as a screening method for particulate matter-exposure assessment and as a complementary method to quantify fungal contamination exposures in indoor environments [10,13].

## 4. Conclusions

Overall, this study allowed us to conclude that EDC should be included in sampling protocols dedicated to performing exposure assessment due to several advantages raised: (a) straightforward extraction protocol obtaining a liquid sample favoring the employment of different assays; (b) possible to be used to assess exposure to a wide range of microbial agents (fungi and metabolites); (c) allows a higher accuracy regarding the fungal diversity when compared with other sampling methods (active and passive); (d) low cost and little disturbance in the workers/occupants´ daily routines that can influence the assessment being performed.

## Data Availability

Data sharing is not applicable to this article as no datasets were generated or analyzed during the current study.
